# A story of data won, data lost and data re-found: the realities of ecological data preservation

**DOI:** 10.3897/BDJ.6.e28073

**Published:** 2018-11-07

**Authors:** Alison Specht, Matthew P. Bolton, Bryn Kingsford, Raymond L. Specht, Lee Belbin

**Affiliations:** 1 University of Queensland, Brisbane, Australia University of Queensland Brisbane Australia; 2 Corymbia Ecospatial Consultants, Canberra, Australia Corymbia Ecospatial Consultants Canberra Australia; 3 Structured Data, Canberra, Australia Structured Data Canberra Australia; 4 Emeritus Professor, Brisbane, Australia Emeritus Professor Brisbane Australia; 5 Atlas of Living Australia, CSIRO, Canberra, Australia Atlas of Living Australia, CSIRO Canberra Australia

**Keywords:** data conservation, data retrieval, legacy data, data curation, long-term data accessibility

## Abstract

This paper discusses the process of retrieval and updating legacy data to allow on-line discovery and delivery. There are many pitfalls of institutional and non-institutional ecological data conservation over the long term. Interruptions to custodianship, old media, lost knowledge and the continuous evolution of species names makes resurrection of old data challenging. We caution against technological arrogance and emphasise the importance of international standards.

We use a case study of a compiled set of continent-wide vegetation survey data for which, although the analyses had been published, the raw data had not. In the original study, publications containing plot data collected from the 1880s onwards had been collected, interpreted, digitised and integrated for the classification of vegetation and analysis of its conservation status across Australia. These compiled data are an extremely valuable national collection that demanded publishing in open, readily accessible online repositories, such as the Terrestrial Ecosystem Research Network (http://www.tern.org.au) and the Atlas of Living Australia (ALA: http://www.ala.org.au), the Australian node of the Global Biodiversity Information Facility (GBIF: http://www.gbif.org). It is hoped that the lessons learnt from this project may trigger a sober review of the value of endangered data, the cost of retrieval and the importance of suitable and timely archiving through the vicissitudes of technological change, so the initial unique collection investment enables multiple re-use in perpetuity.

## Introduction

An argument without evidence is mere assertion (Parsons et al. 2010). Knowledge of change is fundamental to our custodianship of the Earth’s biodiversity. To appreciate and quantify the effects on biodiversity of changes in climate and land use, for example, it is well recognised that we need to call on information from the past and to repeat data collection effort (Lindenmayer and Likens 2009; Jetz et al. 2012; Morris and White 2013; Schimel et al. 2013; Wyborn 2015; Kissling et al. 2017). There are many challenges, however, to realising past data for future use.

The scale of past data collections is often beyond today’s means so replication may be nearly impossible. Cook’s various explorations of the Pacific, Humbolt’s expedition to South America, and Darwin’s voyages in the *Beagle* required great planning, the assembly of many personnel across many disciplines, and occurred over great distances and time. Data-collecting expeditions of similar scale would be prohibitively expensive to launch in modern times (Powney and Isaacs 2015). The wealth of data acquired on such expeditions continues to inform our understanding of the world and how it functions, and it could be argued they are even more valuable in consequence of their very unrepeatability.

These famous expeditions are mere examples of an abundance of organised data collections made over the centuries. We benefit from only a fraction of this knowledge as a huge mass of data from the filing cabinets and the computers of scientists and research teams, despite the best intentions, are poorly described and managed, unavailable, or completely lost (Nordling 2010; Vines et al. 2014). The longer the time-span since an initial collection effort, the harder (and more costly) these data are to retrieve (Vines et al. 2014).

Routine long-term data collection and its ongoing management and conservation is often a low priority in policy-driven government departments, while physical and digital data storage has been increasingly ‘rationalised’ as data custodians have been made redundant and agencies are either downsized, re-structured or abolished (Pickrell 2017; Phillips 2017). Amongst the benefits of archiving data for future use is that new and totally unanticipated uses and value can be found for them. This is well illustrated by the later use of whale catch data collected for taxes and excise duty in the nineteenth and early twentieth centuries for the detection of the effect of climate change in the Southern Ocean (de la Mare 1997). This re-use was only made possible because the data were openly available and fully described. The whalers could never have anticipated that their catch data would be used to detect evidence of climate change. Modern intellectual property laws structured to protect rights to information, however, can discourage or prevent analyses of this nature. Sadly, many data owners are fearful of their data being used for purposes other than those for which it was originally collected (Tenopir et al. 2011; Specht et al. 2015; Mills et al. 2015; Mills et al. 2016). The advent of metadata has at least exposed that data exist, even if they may not currently be publicly available (Bagley 1968). Initiatives such as data carpentry and the integration of mandatory data management plans in research grant applications have increased the acceptance of data publication amongst scientists as something in which they can engage (Teal et al. 2015; Curty et al. 2017). Scientists will always need support in the process of data publication as they need to focus on their primary research (Lynch 2008; Martin et al. 2017).

Recovery of past data is a difficult challenge depending on how the data have been stored (see Specht et al. 2015). Data storage systems have changed profoundly since the beginning of the digital age. Punch cards and paper tape have been superseded by magnetic tape (in a myriad of formats), floppy discs, hard discs, optical discs, flash drives and cloud storage. Even when stored digitally, changes to storage media and formats require continual inspection and potential intervention, without which the data are put at risk of loss (Bergeron 2001; Vines et al. 2014; Michener 2016). Devices that read obsolete media require connection with old cables to old computers and software. Such systems are increasingly hard to find or adapt to modern systems. Even if the media are supported, a data file written in a particular format may not be readable with newer software and, in some cases, even with later version releases of the same software (for example the various versions of Microsoft Excel). Documents written using Wordstar or SuperCalc on 5.25-inch floppy discs using a CP/M operating system (the original documentation for our case study), although stored, are lost for most practical purposes. Some data may need to be recovered from hard copy printouts using, for instance, optical character recognition (OCR), but this recovery process, even if possible, is extremely costly in time and money.

Data may further be broken up across multiple files, in various formats, and may violate basic principles of current best-practice data structures. Although the principles of relational database design were well established by the 1980s (Codd 1970), the computers and tools available to ecologists until recently were often unreliable and expensive or difficult to use (e.g. 1022 on a DEC PDP-10 mainframe).

Data communities (e.g. Data Science Central: https://www.datasciencecentral.com and the Research Data Alliance: https://www.rd-alliance.org), data repositories (e.g. PANGAEA: https://www.pangaea.de; the Australian Antarctic Data Centre: https://data.aad.gov.au; the Knowledge Network for Biodiversity: https://knb.ecoinformatics.org; DRYAD: https://datadryad.org; the Atlas of Living Australia and the Terrestrial Ecosystem Research Network) and data management support initiatives such as DataONE have been developed to facilitate systematic data sharing and long-term data preservation by scientists. Such good intentions will require, however, consistent advocacy and ongoing monitoring.

Ecological data present a particular challenge for management and preservation because they are:

- geographically, taxonomically and temporally unique (Ellison 2010);
- heterogeneous (Reichman et al. 2011; Wieczorek et al. 2012);
- frequently disaggregated and held in the hands of individuals and small organisations (Heidorn 2008).

The heterogeneity of ecological datasets is arguably a consequence of the nature of the profession. A survey of 751 Australian ecologists in 2011 produced more than 160 self-identified sub-categories of ‘ecologist’ (Keniger and Specht 2012) and ecologists typically collect and integrate different types of data simultaneously (Hampton et al. 2013; Garnier et al. 2017). The distance between data collectors and those skilled in delivering the data is considerable (Campbell et al. 2015). We propose that the arduous and costly nature of ecological data collection, reliant on individual effort in remote and often perilous locations, further contributes to a sense of personal ownership of research outputs and a reluctance to share hard-won data.

Although great strides have been made in the past twenty years towards the routine publication of data, properly described, protected and archived for future use, the recovery of past ecological data remains in its infancy. Synthesis centres such as NCEAS, CESAB, sDiv, John Wesley Powell and ACEAS (see www.synthesis-consortium.org) support ecological analyses that only use existing data (Curty et al. 2017). In these centres, small groups of people organise and synthesise existing data for analysis, and release new, cleaned datasets (e.g. Haberle et al. 2014; Sosef et al. 2017). Such work is focussed on defined ranges of data relating to a particular question and, although immensely valuable both for training scientists in data recovery and in the release of datasets that might otherwise have been lost, synthesis groups generally work at a project-by-project level.

We present a case study of a continental set of ecological data that has had a long history of recovery and digitisation: once in the 1980-90s and again this century. Through this example, we illustrate the challenges imposed by changing norms of publication and technology, the benefits of deposition in a curated repository and provide some guidance for data management.

## The original data collection

The chosen case study arose at the dawn of ‘Big Data in Biology’ *sensu* Aronova et al. (2014), when the ability of computers to aggregate and analyse large amounts of data was becoming a reality. A study had been made of the conservation status of vegetation formations across Australia and New Guinea (Specht et al. 1974), using an assessment method developed in Australia (Specht and Cleland 1963; Frankenberg 1971) and adopted by the Conservation Section of the International Biological Programme (Peterken 1967). These assessments were generated through expert opinion, considered appropriate for the time, but were limited by gaps in information and bias. With the advent of mainframe computers by the mid-1970s, an objective approach to the classification of major plant communities became possible, and a grant from the Australian Heritage Commission was obtained for that purpose. Thus, a new project commenced to repeat and update the 1974 assessment taking advantage of the new analytical algorithms, which resulted (inter alia) in the 'Conservation Atlas of Plant Communities in Australia' (Specht et al. 1995).

**Figure 1.**
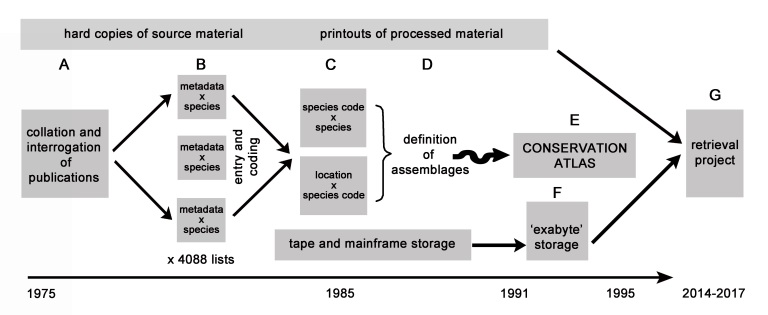
The workflow from collation of original documents (A) through the publication of the ‘Conservation Atlas’ (E) to the retrieval project (G). The first step was to extract and digitise data from written publications (A-B). Due to the computing limitations of the time, it was necessary to split the data into sub-files (B and C) for analysis (D) which was the aim of the original project ('The Conservation Atlas' 1975-1995). Storage throughout the Conservation Atlas project was in both hard copy printouts and digital form. The ‘mainframe’ computers referred to were those from the PDP-10 computer family through the University of Queensland computer centre. The magnetic tapes were used as backup storage from the PDP-10s and the Exabyte tape was used to store the data from the magnetic tapes at the end of the Conservation Atlas project. Note: Letters are used to facilitate reference to the figure from the text. The temporal axis is not to scale.

Published data in refereed journal articles and ‘grey’ literature (i.e. government and research reports) were retrieved in hard copy (Fig. 1A) and full species lists and metadata (vegetation structure, soil type and landscape descriptions) were extracted (Fig. 1B). Partial lists and those without ‘accurate’ geo-references (for the time) were rejected. This was a major task, requiring manual extraction and evaluation by the supervising team and data entry by postgraduate students: 711 ecological surveys incorporating 4088 floristic lists were assembled.

Due to the computational limitations of the time, the data were organised according to vegetation formation (e.g. forests, sclerophyll vegetation, mallee; Specht et al. 1995; Specht and Specht 2013) and each species was given a unique 9-character alphanumeric code to enable data handling and subsequent analysis. These codes necessitated the development of a bespoke system for the creation of two main digital files for each formation: the site metadata (including provenance) with alphanumeric lists of species, and a ‘conversion’ file for the link between the alphanumeric codes and their full scientific names (Fig. 1C). The system for handling the data was standardised in the first three years and the resultant workflow called CAVE (Classification of Australian VEgetation) formed the basis of a procedural manual (Bolton 1985). The last of the raw data were entered and analysed in the early 1990s with species names and metadata information correct at that time. As they were entered, the species lists (with metadata) and analyses were printed for checking and safe-keeping and the data on the PDP-10 family of computers at the University of Queensland were backed up on 9-track magnetic tapes. The compiled data by vegetation formation is shown in Table 1.

Once entered and organised, the data were analysed to define floristic associations using the non-parametric programmes TAXON (Ross 1984) and TWINSPAN (Hill 1979; https://www.ceh.ac.uk/services/decorana-and-twinspan). After validation by experts, a total of 921 major plant assemblages were defined (Fig. 1D). Biogeographic regions were derived from these data using the classification programme PATN (Belbin 1994; http://www.patn.com.au). The distribution of each TWINSPAN assemblage and the biogeographic regions were plotted spatially at 0.5° x 0.5° resolution using Arc-GIS software together with an assessment of the conservation status of each floristic assemblage and published as an Atlas (Specht et al. 1995; Fig. 1E). The original project spanned a period of 20 years (1975 to 1995), involved several scientists and was funded by additional small research grants.

In 1991, when the mainframe computers at the University of Queensland were de-commissioned, the data from four of the five magnetic tapes – only readable on the PDPs – were transferred to exabyte tape, considered the best option at the time. The information on the fifth tape could not be retrieved. The company making Exabyte tapes ceased operations in 2006 (Fig. 1F). Despite attempts at the time, the raw data contributing to this study were not stored digitally.

**Figure 2.**
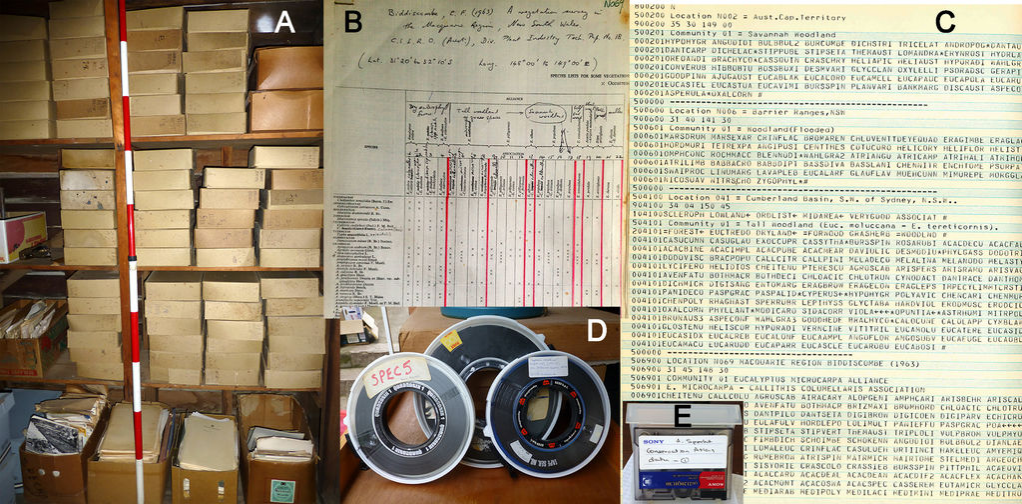
Illustration of the data resources available to the retrieval project: (i) a sample of the boxes of original copies of papers and reports (A), (ii) a table extracted from a publication prepared for data entry (B), (iii) a sample of the hard copy printouts showing alphanumeric lists of species under each location and community (C), (iv) the magnetic tapes on which backups were kept from day to day during the 1980s project (D), and (v) an exabyte tape on to which the data from the magnetic tapes were transferred in 1991 (E).

Physical copies of the original papers, various analyses and data files were stored in Ray Specht’s house when he retired (Fig. 2A-C). The magnetic tapes were stored at Southern Cross University, Lismore, New South Wales, Australia. The ‘Exabyte’ tapes were stored in two locations some thousands of kilometres apart (with A. Specht, who also had the magnetic tapes, and M.P. Bolton) until the present retrieval project commenced in 2014 (Fig. 1G).

## Retrieval

The retrieval project (Fig. 1G) aimed to recover, preserve and deliver the data assembled for the original vegetation assessment project through now-established open biodiversity data repositories. Financial support and some staff time were provided by the Terrestrial Ecosystem Research Network (TERN) and the Atlas of Living Australia (ALA), the two repositories identified as most relevant for these data.

The first challenge was to develop a system for checking and updating the species names at the time of the ‘Conservation Atlas’ data collection. The most efficient and relevant mechanism to do this was through a web-service interface with the ALA (see http://api.ala.org.au, accessed 3 May 2018) which is the relevant authority for Australian species (see https://www.rbg.vic.gov.au/science/projects/taxonomy/atlas-of-living-australia-national-species-lists-project, accessed 11 December 2017).

**Figure 3.**
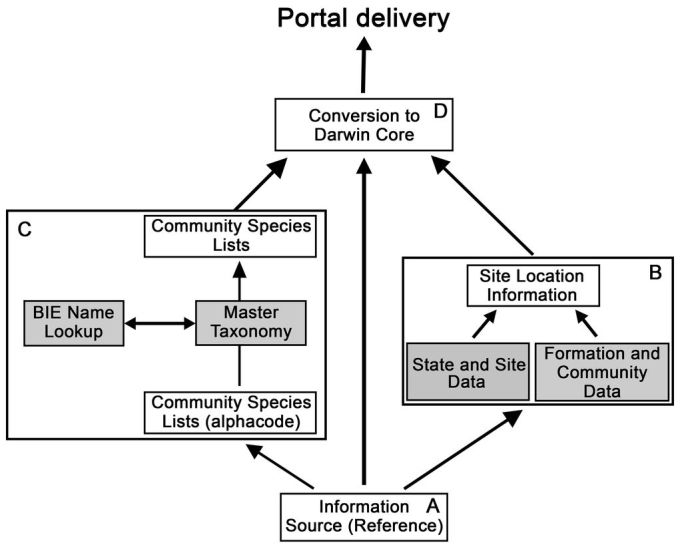
Diagrammatic representation of the workflow for retrieval of data from the original reference files (A). These files were separated into two parts for editing influenced by the 1980s organisation of the data: (i) information on the sites at which data were collected (B), and (ii) the species lists, which were updated through the Biodiversity Information Explorer, BIE (http://bie.ala.org.au/ws) (C). Once these components were updated, they were re-assembled using DarwinCore standards (D) to enable delivery through a data portal (in this case the Knowledge Network for Biocomplexity, KNB (https://knb.ecoinformatics.org). Ecological Metadata Language (EML) was used to describe the dataset.

The plot-based, species structure of the original data was converted to individual observations of species with freely associated data, such as location, date and time, observer, vegetation classification, source and team comments. We wanted to ensure that no information was lost in re-structuring the data for publication using the widely-supported Darwin Core Standard (Wieczorek et al. 2012). The planned process was as follows :

1. **Recover all available data from**
  - Hard copy
  - Exabyte tape
  - Other data in digital form (e.g. Excel spreadsheets) (Fig. 3)
2. **Design a structure that reflects how the data should be viewed from current perspectives** (Fig. 3)
  - Site data/metadata (latitude/longitude by vegetation structure by comments) (Fig. 3B)
  - Species alphanumeric codes and their associated scientific names
  - Sites by species codes (some with multiple communities)
3. **Update the species codes/names to current nomenclature** (Fig. 3C)
  - Use the Atlas of Living Australia’s web services (http://api.ala.org.au, accessed 3 May 2018), the National Species Lists and Australian Plant Census (CHAH: https://www.anbg.gov.au/chah/apc) to semi-automate the current identification of species names
  - Manually check any ambiguous or missing names
4. **Map the fields used in the Conservation Atlas project to the Darwin Core standard** (Fig. 3D)
  - Collate the terms used in the previous studies
  - Determine the intent of the fields
  - Find the best equivalent term in the Darwin Core standard
5. **Collate and integrate the data**
  - Produce a list of species observations using the Darwin Core terms at each site (defined by a consensus latitude/longitude) with metadata including vegetation type/structure, source reference details (Fig. 3A) and processing comments.
6. **Generate a collection-level metadata record.**

The Darwin Core standard (Wieczorek et al. 2012; http://rs.tdwg.org/dwc, accessed 3 May 2018) provides maximum interoperability and is the standard used by the Global Biodiversity Information Facility (GBIF; http://www.gbif.org) and its nodes including the Atlas of Living Australia (ALA). Darwin Core has around 185 fields, more than sufficient to encode the information associated with the Conservation Atlas data. The only other candidate standard applicable to this project would have been Access to Biological Collections Data (ABCD; https://github.com/tdwg/abcd, accessed 3 May 2018), but this standard is far more detailed than required and more applicable to specimen data. Metadata for ecological data are commonly at the collection rather than the record level and the associated standard in wide use is the Ecological Metadata Language (EML; Fegraus et al. 2005). The data were made available through the Knowledge Network of Biocomplexity (see Specht et al. 2018) and are displayed through the Atlas of Living Australia on: https://collections.ala.org.au/public/show/dr8212 (accessed 15 October 2018).

When data retrieval began, the comprehensive computer printouts were the only information source immediately available (Figs 1, 2), as the existence of the Exabyte tapes was unknown. One tape was known to have disappeared (despite having been lodged for safe keeping in the steel cabinet of the GIS office of Southern Cross University where the Atlas was produced in 1995) and the other could not be found. Various hard copy data recovery options were therefore initiated, including Optical Character Recognition. While options were being considered, staff from the Australian Centre for Ecological Analysis and Synthesis (ACEAS-TERN) led by A. Specht, assisted by R.L. Specht, entered the location details from the printouts into a spreadsheet (Microsoft Excel).

A total of 461 locations (135 of these had multiple survey sites within each broad location) were identified from the paper copies and these provided a checklist and structure for the future data compilation. After locating an Exabyte tape reader (not an easy matter either), we found that the tape was fortunately readable but had overlapping content, containing several different file types including basic species and site data, computer programmes for the original data transformation and intermediate and final analysis results (as had the original magnetic tapes and printouts). As noted previously, most of the basic species and site files were consistently structured and were named according to vegetation formation leading to duplication. While confusing, duplication was far preferable to gaps in data. No data remained in either paper or digital form for the rainforest, dry scrubs, alpine vegetation and coastal wetland vegetation formations. This proved to be a loss of a large proportion of the data originally digitised.

Data on 1390 communities were recovered across the remaining formations, with alphanumeric codes for 9450 taxa and associated metadata. The estimated present cost of repeating the collection of raw data from the 461 locations, including species identification, preservation and documentation, would be conservatively AU$29 million. The estimated present cost of extracting and digitising the species lists from the initial articles collated would be around AU$8 million.

## The raw data files

### Core data

The most recent versions of the files were identified relative to the surviving hard copies. The following provides an insight into the complexity of decoding the available files. The digital information was organised (within files by formation) hierarchically: location; source (author); community parameters; and the species codes. Each category was given a control digit to identify the nature of the data following. This provided inputs to (mostly) sequential algorithms programmed in FORTRAN for precise formatting or Pascal for reformatting and quality assurance.

- 800000: an alphanumeric identifier for the state in which the site is found e.g. N for NSW, P for Northern Territory, Q for Queensland etc.
- 50xxxx: location name, unique code (including state identifier) and source.
- 90xxxx: latitude and longitude
- 5xxxbb: community number (bb) at the location, followed by the description
- 30xxxx: additional comments (not always present)
- 00xxxx: a list of 9-character alphanumeric codes for species occurring in the community. The ninth character was reserved for subspecies and varieties, so in most cases was left blank.
- 500000 ------------------------: end of location entry

The fundamental problem with the data format (Table 2) is that the definition and formatting of the data was conditional on the contents of the number block. It was a format optimised for sequential data processing rather than modern approaches, such as "fields" or attributes containing data. This project reformatted the input data into files with .csv formats, but these are far too complex for this paper.

The list of publications, from which the data had been retrieved, was fortunately readable and only required checking and updating. Each citation was given a unique number for the purposes of retrieval (Table 3). Hard copies of most of the source articles that had been obtained for the original project (pre-1995) had been retained so were available to the present authors (Fig. 2). A major component of the retrieval project was to curate the original paper copies, the careful filing of which had been destroyed when R.L. Specht’s papers were removed from his University office when he retired. In consequence, sources that were not or were incompletely digitised at the time of the retrieval project will be more easily able to be scanned and shared with appropriate libraries in the future.

### Metadata

The locations in this project were governed by the historical record (Table 3) and referred to one or more plant community records around a reference point. To provide a checklist for data extraction and to ensure accuracy of translation, a separate ‘master site file’ was created from the hard-copy printouts. This file was referenced to the digital material (source files) as they were found.

The attributes (columns) of the master site file were:

1. Formation: The high-level vegetation classification (Table 1)
2. Source file: The relevant retrieved digital file
3. Line ID Number: Site numerical identifier (if only one community per site, this was the community number)
4. Location number: Alphanumeric code for the location (State code, Table 2)
5. Community number: A sequence number for each community found at the site (1-28)
6. Locality: general description
7. State/Territory
8. Reference Number: The identification number of the associated publication (Table 3)
9. Date of reference to be used if multiple references were cited
10. Latitude and longitude: original
11. Vegetation Type 1 – the broad community description
12. Vegetation Type 2 – additional information such as dominant species or association
13. Comment line number: The line number in the source file containing comments
14. Notes from team(s) attached to the comment line. Some formations had interpretive codes for locations and sites added by the collators (Bolton 1985). These were used as keywords for sub-setting the data and as co-variates in analyses.
15. Decimal latitude and longitude
16. Coordinate uncertainty in metres
17. Comments from retrieval team (using a consistent vocabulary).

Throughout the project there was an evolution of the fields in the master site file. In the original lists, multiple authorities were often cited, with sequential dates, one building on the work of the other or acknowledging a re-citation (an attempt to trace the provenance of a species list). Such multiple citations were not supported in Darwin Core format. Ray and Alison Specht, in consequence, reviewed all multiple author attributions and selected the most relevant to be the primary authority.

The definition of location, now possible with Geographic Positioning Systems (GPS), was not available in most of the original studies. The broad latitude and longitude information in the original datasets (Table 2) had to be updated. This need was exposed when a basic check of the resulting Darwin Core records were entered into the ALA’s ‘sandbox’ (http://sandbox.ala.org.au, accessed 3 May 2018) and mapped in the ALA’s Spatial Portal (see Belbin 2011). All site locations were checked against the original documents where possible and verified using Google maps (satellite view). Decimal degrees columns were inserted and comments made of any amendments to an original location together with an estimate of coordinate uncertainty (Darwin Core term coordinateUncertaintyInMeters).

Several typographical and procedural inconsistencies were highlighted as the datasets were ingested. The duration of the original project — from the first datasets (late 1970s) to the last (early 1990s) — and the splicing of the data into different formations resulted in variations in the way associated information was recorded, from state/territory codes to the numbers associated with record lines for plant communities in the datasets (Table 2). These matters required reference to the original source where possible and updating the data.

When protected species are encountered in the ALA, some of their locations may be obfuscated, resulting in locational refinements being undone. The ALA’s Sensitive Data Service (SDS) examines records of any sensitive species (state, territory, federal or IUCN status) and applies rules depending on the location. As these data are in the public domain, we considered it was justifiable to overrule the SDS.

### Species conversion file

Full taxonomic names are used in most biodiversity information systems and analysis packages (Tokmakoff et al. 2016), and it was central to the goals of this project to turn the original alphanumeric codes into current scientific names. The first step interpreted the alphanumeric codes from the original species conversion files (Rees 2014) and the second step updated the names using the ALA’s web services (Fig. 3C) Species conversion (alphacodes to names) files were developed for each vegetation formation and contained the following attributes:

1. Sequential row number
2. Validity flag: A one-character code
  - L = Legal (Valid) taxon
  - S = Synonym
  - M = Misspelling.
3. Growth habit flag: one-character code based on the eco-morphological attributes listed in Table 3.2 Specht and Specht (2002)
  - A = Aquatic
  - B = Semi-aquatic
  - C = Creeper
  - D = Dwarf shrub (sclerophyllous) <0.25m
  - E = Epiphyte
  - F = Ferns
  - G = Graminoid
  - H = Hummock grass
  - I = Invasive
  - K = non-sclerophyll shrub
  - L = Low tree
  - M = Medium tree
  - P = Parasite
  - S = Shrub >2 m
  - U = Geophytes
  - V = Vine
  - W = Dwarf shrub (non-sclerophyllous) <0.25 m
  - Y = Evergreen
  - Z = Sclerophyllous shrub >0.25 m <2 m
4. A general purpose code ‘G’, indicating stage of analysis.
5. Species code: The 9-character alphacode
6. Scientific name

At the time of the original study, a species name was updated if a new species name was identified. To retain fidelity with the original record, both names were recorded. These updates were performed by R.L. Specht as part of the original CAVE protocol (Bolton 1985, Table 4).

## Digital processing

The datasets in this project were large enough to preclude manual processing. As with the original study, we were therefore dependent on several computer programmes to extract, integrate and validate the data matched to Darwin Core standard terms. This process was facilitated by access to modern programming languages such as Pentaho, Java and JavaScript, utilisation of json format, and ALA web services as noted above.

### Species Names

The largest problem encountered was matching the species names in the data against the National Species Lists. There will always be arguments about species identification and nomenclature. There is no universally agreed taxonomy. This phase took around half of the project programming time, even with recourse to the Australian National Species Lists (http://www.rbg.vic.gov.au/science/projects/taxonomy/atlas-of-living-australia-national-species-lists-project, accessed 26 June 2018). Many names had been superseded over the intervening decades. The 9-digit alphacodes, required for the original TWINSPAN analyses, presented an additional complication, since the codes were guaranteed unique only within each vegetation formation.

In many cases, the original name for the taxon had moved to a third name. In some cases, the original name was again the currently accepted name for the taxon. Splits of broadly-defined taxa e.g. *Acacia aneura* and *Senecio lautus*, into multiple taxa were mostly unresolvable into current names.

Amongst the information returned through this process were the scientific name for the taxon, its globally unique identifier, the taxon concept (essentially the name, named by and named date), common names and a match score. This ALA web service was the key component of the programme that produced a master species spreadsheet containing the best guess scientific name, taxon concept, match type and scores, source files and other parameters. We used five name match categories (Table 5). All results except for ‘MATCH’ had to be manually checked, a laborious task.

This process used online and offline resources in roughly the following priority order, dependent on the nature of the uncertainty:

1. Australian Plant Census (APC: http://www.anbg.gov.au/chah/apc/index.html)
2. Australian Plant Name Index (APNI: https://biodiversity.org.au/nsl/services/apni)
3. Atlas of Living Australia (http://www.ala.org.au)
4. Google (http://www.google.com.au) and Google maps (http://maps.google.com)
5. PlantNET: NSW Flora Online Plant Name Search (http://plantnet.rbgsyd.nsw.gov.au/search/simple.htm)
6. FloraBase: The flora of western Australia (https://florabase.dpaw.wa.gov.au/search/advanced)
7. Australia's Virtual Herbarium (http://avh.chah.org.au)
8. The Plant List (http://www.theplantlist.org)
9. Taxamatch: A programme for matching taxonomic names (http://biodiversity.org.au/service/taxamatch) (Rees 2014)
10. Books and papers (e.g. Brooker and Kleinig 2004, Brooker and Kleinig 2006, Barker 2005, Barlow 1986, Cunningham et al. 1981, Erickson et al. 1979, Harden 1990, Harden 1992, Harden 1993, Harden 2002, Harden and Murray 2000, Jessop 1981, Moore 2005, Nicolle et al. 2012, Orchard 2005, Parsons and Cuthbertson 1992,Stanley and Ross 1983, Stanley and Ross 1986, Stanley and Ross 1989,Tothill and Hacker 1983).

The workflow for name resolution typically followed three stages.

**Stage 1: Current name check**

Often an incorrect name lookup using the ALA web service was caused by the name being misspelled in the original data, sometimes as a result of a simple typographical error. Taxonomists register common mistakes as ‘orth. var.’ and these are registered in APNI (https://biodiversity.org.au/nsl/services/apni).

The ALA name lookup sometimes returned an ambiguous result requiring further investigation. For example, *Eragrostis ciliata* could be mapped to *E. cilianensis, E. ciliolata* or *Ericachne ciliata*. Where only a single letter was used to represent a genus (as was occasionally the case in sequential lists in the digital master species conversion file: Table 4), it was necessary to manually look up the original intended genus. One then needed to go back to the start to see if the name provided a match. The absence of a name on the Australian Plant Census (APC) suggested that the Council of Heads of Australasian Herbaria (CHAH) had not resolved the taxonomy (see http://www.anbg.gov.au/chah/apc/families-treated.html). In such cases, it came down to the best judgement using the above-listed resources.

**Stage 2: Validation**

Validation was dependent on the botanical knowledge of the assessor, in this case primarily Bolton. For the cases of taxonomic splits and misapplied names, additional information was required for name resolution. If no obvious match could be found from the available resources, we checked the original data file. In cases where no clarifying information could be found, the ALA’s ‘Explore your area’ or the ALA’s Spatial Portal (http://spatial.ala.org.au, accessed 26 June 2018) was used to identify potential candidates restricted to one or two of the original sites. Where the sites were associated with a small national park or reserve, the Spatial Portal was used to define the park or reserve as the area of interest and a species list was produced from the area report. Matches were usually found amongst the small number of species in the target genus. A good candidate species was one that was most common and occurred across the park/reserve. This strategy worked well for many taxa in south-western Western Australia.

**Stage 3: Reference to an expert**

Where no obvious species matches could be identified, the list of unmatched names was sent to Ray Specht, the lead author of the 1995 study (Specht et al. 1995) for resolution. Ray made determinations from his knowledge of the flora and the literature sources.

The result of this process was a master species file with 9450 taxa, mostly species names. It would be desirable to link all the species listed in this project to voucher specimens which would potentially enable the several remaining incomplete identifications (to genus, family, sp. aff. etc.) to be resolved. Comprehensively linking these records to vouchers was, however, well beyond the scope of the current project. The voucher specimens will have been deposited in relevant state and national and, possibly, international herbaria. Users may wish to pursue this if necessary and practical for repurposing.

### The final data records

The intention of this data recovery project was to enable the data to be discoverable through as many systems as possible. As the largest challenge was updating the species lists, the resources of the ALA were considered of primary importance. A set of programmes was written to interrogate:

- the master sites file,
- the species conversion files,
- the site x species files, and
- the master publications file

to produce the Darwin Core Records (Fig. 3).

It was not trivial to map the attributes to Darwin Core (DwC). Five main output files were created, each file containing overlapping parts of the DwC Standard, as well as additional data that were not DwC-compliant - either for debugging purposes or because there was no DwC corollary (Fig. 3). The team followed the Completeness model (https://code.google.com/archive/p/ala-dataquality/wikis/CompletenessModel.wiki, accessed 26 June 2018) and used the Darwin Core 'event’ (https://www.gbif.org/darwin-core) to ensure a link to the plot-based approach of the collection. The 47 terms used in the database, including those that had no DwC corollary, are tabulated in the Associated Data files in the KNB repository (Cons_Atlas_DwC_fields_181009.xlsx).

## Discussion

This case study highlights the importance of providing for sustained data curation if we wish to expose data for maximal re-use. The recovery project was started because of the perceived value of the historic data, its national coverage, the fear of complete data loss and the continued existence of the key player in the initial exercise, Ray Specht. The estimated cost of the time the authors have spent in recovering and processing these data is minimally AU$100,000 in addition to the AU$50,000 invested by each of the funding organisations, the ALA and TERN. As a consequence of this effort and commitment, the data are now integrated with the ALA, Australia’s largest repository of species observations (https://collections.ala.org.au/public/show/dr8212) and will, in the future, be delivered as plot-based data through the Eco-informatics facility of TERN. The data set is downloadable from the Knowledge Network for Biocomplexity (Specht et al. 2018; http://doi.org/10.5063/F1QC01QK). We cannot anticipate its possible future utility (de la Mare 1997).

Even though we had access to digital data and supporting materials, a wide range of unanticipated problems were encountered. These should provide a strong warning to those active in or retired from the ecological research community. Many of the problems encountered were the result of:

(a) technological limitations at the time of the initial project and the work therefore required to update the data and formats to suit modern requirements,

(b) changed spatial referencing between the source material and modern standards,

(c) the long time taken to complete the initial project (resulting in variations in formatting and structure of the core data),

(d) the lapse in time between the compilation in 1995 and the start of the retrieval process in 2015 (Fig. 1), and

(e) the evolution of species names.

Changes in species names were expected, but even with the recent digital tools available through the Atlas of Living Australia, bespoke programming and expert taxonomic skills, considerably more time than initially anticipated was needed to resolve ambiguities. Without the effort, expertise and persistence of the authors, the recovery would have been impossible.

The involvement of three people from the original data collection (Specht, Specht and Bolton) in the recovery effort was invaluable for the resolution of taxonomic names, understanding the nature of the overlapping files, interpreting the information recorded, and understanding how the original project had been refined as it developed. Access to the CAVE manual (Bolton 1985) provided descriptions of the various files and fields, and access to the collection of original hard-copy material enabled refinement of the information. Many of the articles referred to were otherwise unavailable either digitally or totally, or could be found only in hard copy, sometimes in only one library in the country. To reproduce the original search and compilation effort would have taken years with delays for article discovery and retrieval, quite apart from re-extraction of the data.

It is interesting to note that there is wide acceptance of the value of the systematic collection of long-term data (e.g. Müller et al. 2010), but such data are rare and often unavailable. Data collection and data collation efforts are frequently spasmodic or at best periodic; the maintenance of continuity and standards remains a challenge. The impetus to collect ‘new’ data with the researcher’s name uniquely attached to it is strong and is fundamental to the training of most scientists. Curation of the datasets of others has not been attractive because such curation is generally inadequately valued. Without adequate evidence across time and space, however, models cannot be built to understand the effects of events like global climate change or changes in our use of the landscape.

In the open-data world, with deposition of data for public use increasingly encouraged and supported through organisations like the Atlas of Living Australia, the Terrestrial Ecosystem Research Network, Elixir (https://www.elixir-europe.org), the Research Data Alliance, DataONE and GBIF, hopefully data loss will be less likely into the future. Even so, scientists need to be trained and encouraged to take advantage of repositories, and sustained funding is required to support the infrastructure necessary for good data conservation outcomes.

The original project was envisioned as a stock-take of the past, and by its conversion to and storage in digital form, a resource for the future. Despite initial enthusiasm for the project, lack of subsequent funding and continuity of effort meant this resource was almost lost. This is a common story even in cases where there was more substantial initial investment (Aronova et al. 2014; Michener 2015).

As our environment and our technological sophistication change, we need to respect information as it was originally reported. An object lesson from this project is not to be scornful of the efforts of times past, but to value them for the information they provide.

Sufficient resources need to be set aside to ensure that:

(a) scientists deposit their data as closely as possible to the time of their creation in appropriate, sustainable digital repositories,

(b) the technology of repositories is updated, and

(c) the data are appropriately conserved, allowing access, while maintaining integrity.

Only thus will data be useful to a myriad of future applications. If not, the cost of recovery of data in the future will be far higher than you may imagine and may, in fact, be impossible.

## Figures and Tables

**Table 1.** Numbers of sites and species in each vegetation formation in the initial project. These numbers include species that occur in more than one vegetation formation. ^*^ = Not including introduced species or singletons within the formation; ^**^ = Not including tree species >10 m tall

**Formation**	**Locations**	**Communities**	Species^*^
Closed forests	n/a	644	1,418
Dry scrubs – SE Queensland	232	232	475
Dry scrubs – Northern Territory	n/a	1,219	559
Eucalypt open-forests and woodlands (tree species)	201	1,275	276
Sclerophyll vegetation SW Western Australia	64	172	1,761
Sclerophyll vegetation Central and Eastern Australia	188	549	2,581^**^
Sclerophyll vegetation – heathland and tall shrubland	136	312	2,071^**^
Alpine vegetation	73	61	556
Savannah understorey	56	198	1,313
Mallee open-scrub	28	41	395
Desert Acacia	54	148	1,229
Chenopod shrubland	30	68	410
Forested wetlands (including brigalow)	31	36	193
Arid wetlands	20	42	642
Freshwater swamp vegetation	80	80	139
Coastal dune vegetation	45	56	315
Coastal wetland vegetation (mangroves and saltmarshes)	n/a	15	74

**Table 2.** An example of the core data available from printouts and (mostly) retrieved from Exabyte tapes according to formation and State. These examples are from the forested wetlands and desert acacia formations in New South Wales (N) and the Northern Territory (P).

**LINE ID**	**Information**
800000	N
503200	LOCATION N032 = CENTRAL COAST: SYDNEY (PIDGEON 1940)
903200	33 51 151 13
503201	COMMUNITY 01 = FRESHWATER RIVER (COMBINED LIST)
003201	UTRIAUST UTRIEXOL UTRIBILO VALLGIGA POTAOCHR POTAPERF POTATRIC BRASSCHR #
003201	NAJAMARI MYRIPROP PHRAAUST ELEOCHAR* TYPHORIE TYPHDOMI TRIGPROC TRIGSTRI #
003201	JUNCPAUC JUNCPALL JUNCPLAN AGROAVEN GAHNIA__* CASUCUNN MELALINA MELASTYP #
003201	CALLSALI EUCAROBU EUCAAMPL CAREX___* ISOLPROL VILLRENI ALISPLAN RANURIVU #
003201	GRATPUBE GOODPANI HYDRPEDU CENTASIA VIOLHEDE PRUNVULG STELFLAC SCHOAPOG #
003201	OPLIIMBE BLECINDI ADIAAETH PHILLANU #
503202	COMMUNITY 02 = FRESHWATER SWAMPS ON WIND BLOWN SAND (PORT STEPHENS)
003202	BAUMTERE BAUMARTI TRIGPROC TRIGSTRI PHILLANU LEPIARTI MELAQUIN EUCAROBU #
003202	ISOLINUN GRATPEDU DROSSPAT VILLRENI BAUMJUNC SCHOBREV RESTAUST LEPTTENA #
003202	RESTTETR SPREINCA BOROPARV EPACOBTU GONOMICR BLECINDI HYDRTRIP SPHAGNUM* #
003202	VIOLHEDE #
500000	-------------------------------
800000	P
503700	LOCATION P037 = TANAMI DESERT: LAKE SURPRISE, N.T. (MACONOCHIE 1973)
903700	20 15 131 45
503701	COMMUNITY 01 = TUSSOCK GRASS-SEDGE-LAND + TREES
303701	EUCAPAPU ACACVICT #
003701	ABUTOTOC ACACADSU ACACJENS ACACMELL ACACSTIP ACACTENU ALTEANGU ARISBROW #
003701	ARISINAE BERGTRIM BONALINE BRACHOLO BRUNAUS2 BULBBARB CANTATTE CASSCOST #
003701	CASSHELM CASSOLIG CASSFILI CLEOVISC CLERFLOR COMESYLV CROTCUNN CROTEREM #
003701	CYPEBULB CYPECUNN CYPEHOLO CYPEIRIA DAMPCAND DESMMUEL DICRLEWE DODOPETI #
003701	ECTRSCHU ELYTSPIC ERAGLANF ERIAARIS ERIABENT EUCAASPE EUCAPRUI EUCASETO #
003701	EUCATERM EULAFULV EUPHDRUM EUPHWHEE GOODAZUR GOODENIA*GOMPCONI GREVJUNC #
003701	GREVWICK HALGSOLA HELIAMBI HIBILEPT HIBISTURC HIBISTURP INDIBREV IPOMMUEL #
003701	ISOTATRO LOMALEUC MARSEXAR MELAGLOM MELALASI MELANERV MELHOBLO MELOMADE #
003701	MERRDAVE MIRBVIMI MORGFLOR NEPTDIMO PANIAUST PARAMUEL PHYLCARP PHYLHUNT #
003701	PHYLRHYT PIMEAMMO PLECPUNG PLUCTETR PLUCTETRT POLYSYNA POLYGALA *PORTFILI #
003701	PORTOLER PSORMART PTILARTH PTILASTR PTILCALO RULILOXO SANTLANC SCAEPARV #
003701	SCIRLAEV SIDAPLAT STACMEGA SWAIBUR3 SYNATILL TINOSMIL TRIAPILO TRIOPUNG #
003701	TRIUGLAU WALTINDI ZORNALBI #
500000	-------------------------------

**Table 3.** Example of records from the publications spreadsheet. ID = our imposed identification number (roughly alphabetical).

**ID**	**Author(s)**	**Date**	**Title**	**Journal etc.**	**Volume No.**	**Page numbers**
1	Abbott, J.	1977	Species richness, turnover and equilibrium in insular floras near Perth, Western Australia.	Aust. J. Bot.	25	193-208
8	Adams, L. D. & Craven, L. A.	1976	Checklist of vascular plants in a study area of the South Coast of N.S.W.	C.S.I.R.O. Land Use Res. Tech. Mem.	76/16	
387	McMahon, A.R.G., Carr, G.W., Todd, J.A. & Race, G.J.	1990	The Conservation Status of Major Plant Communities in Australia: Victoria.	Ecological Horticulture Pty Ltd, Clifton Hill, Vic.		
474	Pye, K.	1982	Morphology and sediments of the Ramsay Bay sand dunes, Hinchinbrook Island, North Queensland.	Proc. R. Soc. Qld	93	31-47
560	Tate, R.	1880	On the geological and botanical features of southern Yorke Peninsula, South Australia.	Trans. R. Soc. S. Aust.	13	112-120
705	Willis, J.H.	1967	Systematic arrangement of vascular plants noted on the slopes and summit of the peak: The Rocks Nature Reserve, New South Wales.	Nat. Pks & Wildl. Serv., N.S.W.	705	

**Table 4.** An example of the species conversion file for the sclerophyll formation and of alphacodes. This example does not illustrate the size of the files.

**Sequential row number**	**Validity and Growth habit flag**	**Species code**	**Scientific name (in publication)**	**New Scientific name (at time of original entry)**
2	L G	ABELMOSC	*Abelmoschus moschatus*	
14	LZG	ACACACAN	*Acacia acanthoclada*	
19	LMG	ACACARGY	*Acacia argyrodendron*	
20	SZG	ACACARMA	*Acacia armata*	*Acacia paradoxa*
21	MLG	ACACASHA	*Acacia ashanesii*	*Acacia oshanesii*
174	S G	ACACKEMP	*Acacia* sp. aff. *A. sibirica*	*Acacia* sp. aff. *A. kempeana*
466	S G	BORRCARP/	*Borreria* sp. aff. *Carpentariae*	Spermacoce sp. aff. stenophylla
704	S G	CARPAEQU	*Carpobrotus aequilaterus*	*Carpobrotus modestus*
705	L G	CARPMODE	*Carpobrotus modestus*	
3019	SIG	RUMEACET	*Rumex acetosella*	*Acetosella vulgaris* sens. lat. *
3020	SIG	RUMEANGI	*Rumex angiocarpus*	*Acetosella vulgaris* sens. lat. *
3647	S G	ZYGOFRUT	*Zygophyllum fruticulosum*	*Zygophyllum aurantiacum*
3650	L G	ZYGOIODO	*Zygophyllum iodocarpum*	

**Table 5.** Species name match categories.

CODE	Meaning	action
MATCH	Near-exact match or better	accept
PARTIAL-L and PARTIAL-R	A significant substring match	manual check
FUZZY	Fuzzy matching algorithm built on the score from the web service using a 'letter-pair similarity' score	manual check
WEAK	A weak match falling below thresholds; the best match is retained	manual check
TAXM	No match or major problem with original or subsequent species name	refer to expert
